# Lived experiences of motherhood among students in a university in KwaZulu-Natal, South Africa

**DOI:** 10.4102/phcfm.v17i1.4757

**Published:** 2025-04-30

**Authors:** Lungisile Shange, Pranitha Maharaj

**Affiliations:** 1 Department of Population Studies and Demography, Faculty of Humanities, North-West University, Mahikeng, South Africa; 2 Research Entity: Social Transformation, Faculty of Humanities, North-West University, Mahikeng, South Africa; 3 School of Built Environment and Development Studies, Faculty of Humanities, University of KwaZulu-Natal, Durban, South Africa

**Keywords:** early pregnancy, early childbearing, student mothers, university, South Africa

## Abstract

**Background:**

Despite ongoing attempts to minimise pregnancies at a young age, early childbearing continues to be one of the world’s most pressing social concerns. South Africa is not immune to this problem, with many young females becoming mothers at an early age. Although South Africa’s fertility rate has decreased over time, the prevalence of early childbearing remains high.

**Aim:**

This study explores the experiences and perspectives of young mothers who are university students.

**Setting:**

The study was conducted in KwaZulu-Natal, South Africa.

**Methods:**

This was a qualitative study and employed an exploratory research design. In-depth interviews were conducted with 20 African women, aged 18–24 years, to learn about the challenges that young mothers face daily and how this affects their academic careers.

**Results:**

The mothers indicated that it was challenging to balance parenthood with their academic pursuits because both are incredibly demanding. As a result, the student mothers’ academic performance suffers, reducing their future earning potential. Most young mothers were single and unemployed, so they relied on their parents to care for them and their children. Almost all the mothers reported limited assistance for young mothers at the university.

**Conclusion:**

A national education policy should require universities to adopt resources or specifically designed programmes to promote better support for student mothers.

**Contribution:**

This study highlights the need for a better understanding of, and continued research into the type of support structures that are available for student mothers.

## Background

Despite ongoing attempts to minimise early pregnancies, early childbearing continues to be one of the world’s most pressing social concerns. Early childbearing refers to first births occurring before a woman is physically and emotionally ready to have a child.^1^ Early childbearing refers to pregnancies occurring at a very young age, that is before the age of 18.^2^ As of 2019, teenagers aged 15–19 years in low- and middle-income countries had an estimated 21 million pregnancies each year, with nearly 50% being unplanned and resulting in an estimated 12 million births.^3^ Student pregnancy is said to be a worldwide issue, but the problem is obvious in developing nations like South Africa.^4^ According to Dlamini-Zuma,^5^ South Africa continues to have the highest rate of adolescent pregnancy in the world, with roughly one in every four girls becoming pregnant before the age of 20.

Early childbearing is problematic as it can affect young women’s educational attainment and, in some situations, leads to school dropout, particularly in developing countries.^6^ Pregnancy and parenting in universities are a global problem as the number of pregnancies among university students is increasing worldwide.^7^ In some countries, student mothers at universities are considered unusual,^8^ but^9^ there was a reported increase in the number of female students at universities, some of whom are mothers.

Although gender inequities in education have been minimised, little attention has been paid to the issues faced by student mothers on campus.^10^ Research shows that the number of universities that provide on-campus childcare has fallen, while demand has increased.^11^ While chances for women to study at university have grown compared to previous generations, student mothers, in particular, are presented with the additional burden of parenting, in addition to their academic work.^12^

Managing academic and parenting commitments can be tough, especially because the academic community prioritises accomplishment and competition above providing adequate support.^13^ Malatji et al.^14^ stated that university administration and personnel lack the necessary abilities to help pregnant and parenting students in returning to academic pursuits following childbirth. However, Wekullo and Isna,^15^ claimed that universities are gradually recognising and addressing this particular set of students but rely on general statistics intended for policy experts.

According to Gault et al.^11^ many women balance university and parenting on their own, often without the aid of their partners. Raising a child while attending university is one of the most difficult barriers to a student’s ability to complete a university degree, necessitating a complex balancing act between family, employment and university responsibilities.^11^ It is difficult to be a student and a mother without compromising one’s activities.^8^

In 2017, there were 3261 births in South Africa among girls aged 10–14 years, 119 645 births among girls aged 15–19 years and 244 190 births among girls aged 20–24 years. Research on early childbearing has focused mainly on high school mothers rather than university student mothers. Studies that focused on university students generally concentrated on part-time students, which typically provided insufficient information about the mothers; others focused on older student mothers. Although some young women become mothers while attending university, many do so before enrolling.

The truth is that many young women at universities have children. It is crucial to understand the difficulties that university student mothers encounter in managing their education and parenting to identify solutions to help them avoid falling behind in their academic performance. This could help institutions improve how they manage difficulties that are special to student mothers. The purpose of this study was to provide insight into the viewpoints and experiences of young mothers who are enrolled as full-time university students.

## Research methods and design

### Study design

The study employed an exploratory research design. According to Drew (2023),^18^ exploratory research is a research methodology that is employed when researchers wish to obtain a more comprehensive understanding of a subject and have a limited understanding of it. It was necessary to conduct this study because, as far as the researchers of this study are aware, no previous research in KwaZulu-Natal has focused on the lived experiences of young mothers who are also university students. By using semi-structured in-depth interviews, the study was able to provide a deeper understanding of the topic. The study was free of bias. As none of the participants were acquainted with the researcher, their opinions were unaffected in anyway. The interview schedule was devoid of leading questions, instead, the participants responses were clarified through probing. Additionally, the researcher established a relaxed atmosphere for the participants so they could freely express themselves and provide honest answers.

### Study setting

The study was conducted in KwaZulu-Natal, South Africa. KwaZulu-Natal has a population of around 12.42 million people, the bulk of whom are African. The study site was chosen because the province has one of the highest youth fertility rates in the country.^16^

### Population and sampling

At the time of the interviews, all respondents were mothers who lived in Durban with their children. The study included solely African women who were between the ages of 18 and 24 years old. Research shows that African girls had a higher rate of early childbearing than other ethnic groups in South Africa.^19^ This study included only student mothers who lived with their children, not those who lived in university dormitories. Six of the young mothers were married or cohabiting, while fourteen were single. Eleven were postgraduates and nine were undergraduates. Because of the sensitivity of the subject of motherhood, non-probability sampling procedures, namely, snowballing and convenient sampling, were utilised to select young African women who were willing to participate and discuss this private element of their lives. Participants were invited to the interview through referrals from other participants. The researcher then approached them directly and asked if they would be interested in participating in the study. The researcher then made appointments for interviews with those who consented to participate in the study.

### Data collection

In-depth interviews (face-to-face) were conducted in 2016 with 20 African student mothers to determine the challenges they experience in their everyday lives and how these affect their academic careers. Participants were recruited from a variety of sources, mostly by snowballing from one participant to the next, to ensure that the sample included a diverse spectrum of young mothers. Participants were also recruited utilising convenient sampling. The first author of this article, an African female of the same age as the young mothers, conducted all the interviews and led the conversations using a semi-structured interview guide. For convenience, the interviews took place at the homes of the mothers. After explaining the purpose of the study and the fact that participation is completely voluntary, the researcher read the consent form to the participants. Following their reading of the consent form, participants were given the consent form to sign after confirming that they understood the purpose of the study and were willing to participate in the study. All interviews were digitally recorded with the mothers’ permission and then transcribed verbatim for thematic analysis. Recognising that not all participants would be able to articulate properly in English, the researcher created two interview schedules, one in English and one in isiZulu. The interviews conducted in IsiZulu were later translated into English for data analysis. The interviews conducted in IsiZulu were translated by the first author, whose native language is also isiZulu. Each interview lasted for about 15–20 min. Each interview began with a demographic description and reproductive history, followed by questions about their experiences as student mothers, reactions to pregnancy, the impact on their academic performance and future goals.

### Data analysis

Data were analysed and coded using thematic analysis. According to Alhojailan,^20^ thematic analysis is used to analyse classifications and present themes or patterns related to the data. This helped the researcher to obtain a comprehensive, detailed and complex description of the data on what was heard during the interviews. The researcher familiarised herself with the data, delving into the data through repeated readings, looking for meaning and patterns before coding it. When the initial codes were created, the data were coded into meaningful groups. In addition, the data were sorted into different codes according to potential themes and all relevant coded data excerpts were compiled within the identified themes (theme search). The themes were then refined and renewed by reading all the compiled excerpts for each theme and checking whether they appeared to form a coherent pattern.

### Ethical considerations

The study was conducted in compliance with the Declaration of Helsinki and involved human subjects. After agreeing to participate in the study, the participants were given a consent form to sign to prove that they willingly consented to engage in the study and understood what the study entailed. Pseudonyms, instead of the names of participants, were employed to maintain confidentiality and anonymity. The study was approved by the University of KwaZulu-Natal’s Human and Social Science Research Committee on 11 March 2016. The ethical clearance number is HSS/1641/015M.

## Results

This study sought to understand the perspectives and experiences of university students who were also mothers. Interviews were held with 20 student mothers. Information was obtained on a number of themes including becoming a student mother, the impact of motherhood on academic performance, aspirations for the future and compensation for disappointing their parents.

### Becoming a student mother

Many mothers indicated that being a student mother was the most difficult experience they had ever had. Taking on the responsibilities of parenthood and academic work was extremely difficult and demanded a high level of dedication. While they acknowledged the fact that they had faced numerous challenges, they also valued their experiences as mothers. They acknowledged the benefits of motherhood, as well as the feelings of joy and fulfilment that come with having children. The mothers said that motherhood gave significance to their lives and made them feel like they had a purpose.

‘I enjoy being a mother, but it is not without its challenges. Sometimes I feel like I’m not coping, especially with academic work, but the child is already here, and I need to be strong for him.’ (Philile, 22-years-old, female, student)

Most mothers reported that having a child altered their priorities and perceptions of life, with the transition to motherhood followed by personal growth. They said that since becoming mothers, they had grown more caring and less selfish. Some claimed that parenting had made them wiser, and they found themselves maturing faster than their friends. Furthermore, they learned to be more responsible, which included putting their children’s needs first and learning that the world does not revolve solely around their own. These events strengthened their focus and determination to achieve.

‘I learnt to be more responsible, and I began to see things and life differently. I believe I matured emotionally as well.’ (Mbali, 24-years-old, female, student)

Some stated that as mothers, they had to make compromises, and that the pressures of academic work and motherhood left little time for socialising, causing them to lose some friendships. They noted that their friends who did not have children struggled to grasp their new role as mothers. The mothers mentioned that they had extremely hectic schedules, making it impossible for them to spend time with friends or participate in social events. They reported that after having children, they could not come and go as they liked, as going out required them to find someone to look after their children, and they had to have a solid reason for doing so.

‘I am unable to perform tasks that are typical of 23-year-olds. It is difficult for me to leave the house since I must care for my children. This has had a significant impact on my social life because so much has changed.’ (Nonku, 23-years-old, female, student)

### Facing the inevitable

The young mothers noted that there are some things they cannot prevent, such as their child becoming ill, disturbed sleep because of the infant crying nonstop and breastfeeding in the middle of the night. They noted physical tiredness, sleepless nights and emotional problems because of the disruption to their sleeping routines. They stated that they are sometimes compelled to skip lectures because the child is ill, and they need to take them to the clinic.

‘My mother watches after my kid while I am at school, but when she becomes ill, it is my obligation to take her to the clinic. I mean, I already feel horrible that she looks after my baby; I can’t ask her to take her to the doctor unless I have a test or exam to take.’ (Nonku, 23-years-old, female, student)

Some mothers who could afford nannies complained that their nannies are not always dependable. They stated that sometimes they do not come to work or disclose their absence, allowing them to make plans for their children. Furthermore, the mothers stated that nannies occasionally arrive late for work, and if they have early lectures, they are always obliged to skip them as they cannot leave the baby alone unsupervised. Although many participants believed that sending their babies to daycare or pre-school was the best alternative, they were concerned that it was too expensive and that nannies were a more affordable option.

‘Pre-primary school costs around R2300 per month for one child, and I have two. My children have a nanny; however, it is difficult for me when she does not come to work and does not notify me in advance because I now must miss classes to care for the children.’ (Thando, 23-years-old, female, student)

### Impact of motherhood on academic performance

Most mothers reported that parenthood had a negative impact on their academic career as they did not have enough time to focus on their education. They indicated that they were unable to complete their academic work at home because their children demanded their full attention. When they are on campus, they rarely have enough time to complete all their academic obligations because, after attending lectures, they only have a few hours to complete their assignments before returning home. Most mothers stated that they had to leave campus early to avoid large queues at the taxi rank or bus stop in the afternoon. They were unable to focus on their academic work at home because of numerous interruptions, such as household duties and childcare activities. Some mothers stated that the only time they could focus on their academic work was when their children were sleeping. However, they also stated that they are sometimes unable to complete their assignments because their children have kept them awake all night, and that they can only focus on their academic work when their children are asleep.

‘I cannot study at home because one of them pulls me this way and the other pulls me that way. I cannot concentrate at home because everyone wants my attention. Obviously, I must come to school, even at night, because we have so much homework. When I had them, it was tough since I wasn’t used to having a child while also having schoolwork, so work piled up because I was in second year and was supposed to breastfeed. As a result, I did not do particularly well.’ (Thando, 23-years-old, female, student)

The mothers also revealed that parenting had a negative impact on their academic performance, which had deteriorated dramatically, with several mothers reporting large drops in grades. Some young mothers said that they were unable to complete their studies within the expected time for the degree because it was difficult for them to balance the demands of both their education and parenting. They also reported elevated stress levels while their children were ill, emphasising how difficult it was to focus on studies when the children were in discomfort because of sickness.

‘Initially, it had an impact on my academic achievement because I was taking eight credit modules and failed two because it was so difficult to manage. Everything is demanding. Being a mother is demanding due to the amount of care required. It is demanding in every manner; therefore, you usually lose weight; and education is equally demanding.’ (Thando, 23-years-old, female, student)

The mothers stated that money is an important factor in raising a child, noting that it is costly and requires financial stability. They stated that when they cannot afford to meet their child’s fundamental demands, their level of stress increased. Most of them were unemployed and relied on their families for financial support, albeit not all came from financially solid homes. Mothers from financially secure homes reported that their performance was unaffected as they did not have to worry about money, which was already taken care of by their parents.

‘My parents do everything for my child; it’s as if I’m coparenting with them. I consider myself fortunate because I never have to worry about the cost of my baby’s formula or clothing. My academic performance has not deteriorated significantly because my parents are very supportive, and when I have homework or tests, my mother cares for my baby.’ (Pearl, 23-years-old, female, student)

The mothers from low-income families reported receiving little help from their family and having to look for part-time work to care for their children. This made it nearly hard for them to balance their university work, parenting and careers at the same time, as they had too much on their plates and ended up ignoring their academic work, which had a detrimental impact on their academic achievement.

‘Juggling parenting, academics, and a part-time job is difficult. I am a waitress who works afternoon hours and usually gets home around 12 in the morning. By the time I get home I am exhausted and all I want to do is go straight to bed. I never have enough time to complete my academic work. I sleep for about three to four hours before waking up to prepare for school. As a result, my academic performance has deteriorated.’ (Andile, 24-years-old, female, student)

The mothers pointed out that it is especially difficult when the fathers refuse to take responsibility of their children, forcing them to do everything themselves. Some mothers stated that they did receive help from their fathers, but that if there was a problem or an argument, he would punish them by removing child support.

‘My child’s father provides everything for my baby, but when we get into a quarrel, he withdraws child support. It is extremely irritating because I am unemployed, and he knows he is the sole source of income for my baby.’ (Nonku, 23-years-old, female, student)

One mother stated that her parents care for her children, but they no longer assist her financially because they are already caring for her children. She stated that even with her parents’ assistance, she feels forced to contribute, with the little money she earns going toward her children’s necessities. She stated that her parents are financially secure and that they have hired a nanny to care for the children while she is at school, but that once she returns home, she must assume full responsibility for them. Although she does not have to worry about money, her academic performance suffers because she is completely responsible for her children when she returns home and is thus unable to study successfully.

‘I went from being a cool kid with money, the latest phone, and everything to wearing old clothing for R5. I am not going to lie; it had a big impact on me because everything I earn now goes to my children. I mean, I need to think about formula, nappies, and care in case they get sick. I mean, kids do not consume everything grownups eat. I must prioritise their needs before myself. Of course, I would occasionally sacrifice my hair. You see, my hair is so natural right now.’ (Thando, 23-years-old, female, student)

### Support for student mothers

Most mothers were unmarried and unemployed, so they lived with their parents, who provided financial and emotional support. The grandmothers’ support was crucial in assisting the mothers to stay in university and complete their academic duties. The mothers indicated that the grandparents played various roles. The grandfathers supported them and their children financially, while the grandmothers provided emotional support and cared for their children while the mothers were in class. The mothers revealed that raising children while studying is possible provided the mother is supported by her family. Many mothers reported that with their mother’s help, they were able to concentrate on their academic work. It is crucial to emphasise that not all mothers in this study receive support from their families; therefore their academic work was not prioritised, and their performance was badly impacted. Some mothers in this study did not live with their parents for different reasons, including marriage and expulsion from their homes after their parents found out about their pregnancy. The mothers who were expelled from their homes and those who were married stated that it was extremely difficult for them to focus on their studies because as soon as they arrived home, they had to devote all their time to their children and household chores, leaving them with insufficient time to complete their academic work at home.

‘I am very lucky to have supportive parents, especially my mother. Yes, my father provides financial support, but my mother does the most. When I have assignments and exams, she looks after my baby to make sure that I do not get disturbed. Even when I am at school, she is the one who looks after my baby.’ (Pearl, 23-years-old, female, student)

‘Unfortunately for me, when my parents found out that I was pregnant, they kicked me out of the house, so I moved in with my child’s father’s family because I had nowhere else to go. They try to be supportive, especially my child’s grandmother but because I know they are not my family, I make sure that when I return from campus, I do anything I can to help around the house because I feel guilty that they’ve been caring for my baby all day while I was at school. So, as soon as I get home, my child is completely my responsibility.’ (Zodwa, 21-years-old, female, student)

Most mothers reported that they struggle academically because they are unsupported by the university. They noted that no programmes are explicitly designed to support student mothers. Some mothers complained that certain lecturers were exceedingly harsh on them, lacked compassion and understanding and failed to understand their circumstances, resulting in underperformance.

‘One of my professors was constantly criticising me for being a mother. She always brought it up so that everyone knew I was a mother, and she effectively discriminated against me in class because I was a mother.’ (Thando, 23-years-old, female, student)

‘There are no programs for student mothers. I’ve never seen or heard of them. I believe that some lecturers intentionally complicate the lives of mothers. I am married, and having children is natural and expected, yet one of my lecturers gave me hell. She didn’t even bother to understand what was going on. I am already overburdened, and my obligations are difficult and demanding enough, but my lecturer refused to budge. I ended up failing the module and am now repeating it.’ (Sindi, 24-years-old, female, student)

### Aspirations for the future

Many mothers claimed that having children inspired them to work hard and be more determined to attain their goals. Being a mother made them more focused and career-oriented, with the desire to offer a better future for their children driving them to success. They said that because raising children was difficult, it was essential to have a career, and that education was their passport to higher-paying jobs.

‘Once you become a mother, you must ensure that your child gets all that he requires. That is why it is critical to obtain a degree so that you may secure a good career and provide for your child. When you have a child, you realise that it is no longer just about you and what you need, but about the person you have brought into this world.’ (Thulile, 24-years-old, female, student)

Most mothers emphasised that the best way to care for their children was to obtain a university qualification, as this would open doors to employment, with better financial prospects for them. They stated that being a mother leaves no room for selfishness, particularly when it comes to their children. They mentioned that as mothers, they felt compelled to continually compromise to provide their children with everything they believed they deserved. The mothers further stated that acquiring a degree was important for them to strengthen their financial independence, but also to set a good example for their children, demonstrating that with education, anything is possible.

### Compensation for disappointing parents

Some mothers explained that having children at a young age and while still in university strained their relationship with their parents, particularly because their parents expected them to finish school and find jobs before considering having children. These mothers said that they wanted to make it their mission to complete university to show their parents that their aspirations were still important to them and that the dreams that their parents had for them were still attainable. Some mothers from underprivileged families reported that their parents were very disappointed in them because they hoped that they would finish university and change their family’s situation.

‘You disappointed me, my child; we were counting on you to change the situation at home.’ (Zinzi, 22 years old)

‘You are aware of our financial predicament, but you chose to become pregnant before finishing university. How will we raise this child because you know very well that we are struggling financially? Where will the money for raising this child come from?.’ (Zinhle, 20-years-old, female, student)

Some mothers said that knowing that they had disappointed their parents was stressful for them as they knew they had to work hard in school to pass because they could not let them down again by failing. They also stated that they wanted to show their parents how grateful they were that they enabled them to continue with school and did not force them to look for work so that they could support their children. One mother claimed that after giving birth, she was having difficulty keeping up with academic work and ended up failing some modules, and her mother said to her:

‘If you hadn’t chosen to get pregnant while still studying, you wouldn’t have failed.’ (Noxolo, 19-years-old, female, student)

Some mothers said that being a student and a mother is already difficult, and now on top of that they stress about not disappointing their parents again by failing at school because they doubt their parents will continue to support them. Despite everything, they still wanted to make their parents proud and restore their relationship.

In summary, the results showed that having a child while studying can negatively impact the academic progress of student mothers, although this is not the case with all mothers. Through sufficient support from the immediate family and the children’s fathers, the workload becomes manageable. Unfortunately, not all mothers have a good support system, so those who do not have it are mostly negatively affected. Despite the challenges faced by student mothers, they expressed that they still love and enjoy motherhood and that their pursuit of an education is primarily motivated by the ability to care for their children.

## Discussion

The aim of this study was to shed insights into the experiences and perspectives of young mothers who are also university students by drawing on qualitative interviews. The interviews allowed the young women to share their experiences of motherhood. The interviews demonstrated that young mothers face substantial obstacles in managing school, parenthood and, in some circumstances, work. One study revealed that student mothers often feel excluded from mainstream academia because of their perceived incapacity to compete with peers.^13^ Lyonette et al.^17^ suggested that recognising student parents’ particular needs and providing specialised support is crucial to preventing them from dropping out. One of the key issues raised by the mothers in this study was a lack of support from the university and its lecturers. In a different study, student mothers complained that professors are not supportive and do not understand their unique demands.^21^ One mother in this study recalled having some negative encounters with one of her lecturers, who would go out of her way to attempt to shame her in front of the entire class, just because she is a mother. The lack of support from the universities and their staff members appears to be a prevalent issue, as it was also observed in other studies.^12,17,21,22^ Although many mothers in this study stated that they do not expect special treatment simply because they are mothers, they do wish that there were programmes expressly geared to assist student mothers in coping better with their academic pursuits at universities. However, participants in one study claimed that they do not seek help from the university because they believed that it was not the responsibility of the university to deal with their personal stories and struggles.^22^

The results indicate that the most difficult aspect of being a student mother is balancing parenting and academic work at the same time, with family duties interfering with academic performance. Dankyi et al.^23^ found that student mothers had to skip certain lectures because of their hectic schedules, and that they occasionally needed to attend to other non-university-related tasks. According to Ngum et al.^24^ young mothers struggle to cope with the additional duties of motherhood, however Taukeni^25^ indicated that this is because of student mothers’ inability to balance both roles. Similar findings were reported in a South African study, which found that student mothers’ complications were exacerbated by the fact that those who live with their children had to divide their time between travelling to and from university and making sure that their children arrived at creche and school on time.^22^ These results are in line with the study’s findings. In a study conducted with university student mothers in the United Arab Emirates, mothers reported being unable to complete academic work at home because of high demand and a desire to prioritise their young children’s needs.^26^ This demonstrates that it is not only South African moms who struggle to balance both duties; it also occurs in other areas of the world. Student mothers have numerous problems that complicate their academic efforts, some of which are inevitable.

The current study found that student mothers’ busy schedules had a negative impact on their academic progress. These results are consistent with those of the study conducted in Kenya, where student mothers reported lower educational attainment because of the difficulty of balancing school and parental obligations.^27^ In this current study, some student mothers reported failing various modules because they were unable to balance motherhood and education, as well as split their time effectively between caring for their children and focusing on their academic work. In an earlier study,^28^ a mother said that she has little free time because of multiple commitments and a need to prioritise her child’s well-being. One of the reasons that contributed to poor performance by student mothers was the inability to attend classes or submit assignments on time. Additionally, the mothers stated that they had to miss lectures because either no one was available to look after the baby or the child was ill and required hospitalisation. Research conducted with student mothers in Ghana revealed comparable findings.^23^ This could be because of a lack of support from family members or because family members expect more from mothers in addition to their maternity responsibilities. For example, some mothers expressed that they are still expected to come home from school and do household chores in addition to caring for their children.

The mothers expressed that they appreciate friendships but struggle to maintain them as their friends who are not mothers sometimes fail to comprehend their new roles as mothers. Given that these are young women who lived a different life before having children, it is understandable that their peers’ attitudes about them shifted because of their varied responsibilities and priorities. In a study conducted in Iran, a mother revealed that when she goes out with her friends, she always takes her child with her, and the presence of the child makes her friends uncomfortable.^28^ The mothers stated that before they had children, they led a different life, occasionally spending time with their friends and doing things that they can no longer do now that they are parents. In a study by Taukeni^25^ the mothers mentioned that becoming a mother is difficult because one can lose friends, and in other cases, friends refuse to walk with them. This suggested that their friends felt uncomfortable to be seen with them solely because they are mothers. Although most mothers felt lonely as they had no friends and spent too much time caring for their children and studying, they used this as motivation to work hard and concentrate on their studies. This study, however, discovered that friends gradually start to distance themselves from the mothers because their priorities have changed or moved, and they are no longer interested in or have time to do the things they used to do before having children.

Raising a child and attending university are both expensive, making it nearly hard to achieve these two goals effectively without money. Single student mothers face the greatest risk of unfavourable outcomes, both in terms of the expectations placed on them and in terms of financial difficulty.^17^ It has already been established that the mothers in this study are young women, and most of them are unable to financially support their children because of them being unemployed. Financial stability is crucial not just for the benefit of the child, but also because money is still required to successfully complete a university degree. Maisela and Ross^22^ argued that while some student mothers are married and have financial and emotional support from their partners and families, others may be single parents without such assistance. Financial difficulties were found to lead to poor academic performance.^29^ Financial troubles can cause stress and anxiety, prompting women to seek alternative sources of income. One mother stated that when she is concerned about not having enough money to feed her child or buy the things her child needs, it is tough to concentrate on her academic studies. Research indicates that when students’ financial position became unsustainable, they temporarily abandoned various educational activities, such as lectures, in order to raise funds for essentials.^29^ Many mothers in this study stated that they depend heavily on their parents for financial support, especially in cases where the fathers are absent or do not provide any financial support. Similar findings were obtained in a study in the mothers in this study who praised their parents for genuinely being parents to their children and providing for them in ways that they are unable to, especially financially. This was also reported in a study by Lyonette et al.^17^

Although several mothers underlined the difficulty of managing parenting and school, many also stated that with the right support, it is feasible. Many mothers in this study mentioned their parents as their support system, which made their academic journey bearable. Family support, whether financial or emotional was found to be very important and lessened the burden of having to care for a child alone while attending university and promotes resilience among student mothers.^12^ Almost all the mothers stated that having children is one of the motivators for them to complete their studies, not only to be financially secure, but also to demonstrate to their children that no matter what the situation is, if a person works hard, they can achieve anything they desire. One study shared similar findings where student mothers reported that they saw motherhood as a drive to finish their studies and a pathway to a better future for them and their children^12^ According to, Bosch^31^ student mothers continue their tertiary education because it provides a sense of personal achievement and self-development, as well as an opportunity to better their professional identity and self-confidence.

### Strengths and limitations

This study was not without limitations. Although numerous academic studies exist concerning early childbearing, few specifically focus on university students, particularly in South Africa. Moreover, the limited literature accessible is often outdated. In relation to data collection, recruiting participants posed challenges because, despite employing snowball sampling, not all of the women to whom we were referred consented to participate in the study. Several participants expressed a desire to exit the interview prematurely because of concerns that others might uncover who disclosed specific information. Consequently, persuading them regarding the confidentiality of the interviews became a challenging task. However, revisiting the consent form with them and assuring them that all statements made during the interview will remain confidential between them and the researcher influenced their final decision to participate in the study.

This research succeeded in delivering a comprehensive understanding of the experiences of the student mothers. It highlights the need for a better understanding of, and continued research into the type of support structures that are available for student mothers. The sample size was a suitable representation of the group under investigation, considering that the participants were drawn from various areas within the study location and were not from the same community. This is a highly significant subject, and employing qualitative research to reveal their perspectives and experiences was more appropriate.

### Recommendations

The finding additionally indicated that student mothers are experiencing academic difficulties because of a lack of support from the university. It is not the responsibility of the university to facilitate an easier life for student mothers. Nonetheless, some assistance from the university would be greatly beneficial. It is the obligation of every student to ensure they seek avenues for enhancing their academic experiences, irrespective of their status as mothers. Given that a significant number of student mothers reported encountering academic struggles, they can assist themselves by establishing their own support group, such as a support group for student mothers, where they can aid one another. This support group would be advantageous as student mothers would have the opportunity to discuss the challenges they encounter as mothers and also collaborate on academic work. Universities should play a role in promoting these support groups for student mothers as a means of demonstrating support. If universities are involved, students will take the support group seriously and it will be more convenient for them to participate. A support group for student mothers could assist these students in excelling academically.

There exists a necessity for a policy that rigorously promotes the academic wellbeing of student mothers, and all universities, or institutions of higher education, should implement this policy to assist these mothers, as their academic needs may differ from those of students who are not mothers.

## Conclusion

Student mothers confront unique obstacles that differ from those faced by other students, and they should be addressed accordingly. They receive little help from universities, and as a result, their academic performance suffers. As most student mothers have stated that they are struggling academically, they can help themselves by forming their own support groups where they can assist one another, be it academically or emotionally. This support group would be beneficial as student mothers would be able to discuss the difficulties they confront as mothers, while also assisting one another with academic work. Universities should help to promote these support groups by listing them on student sites so that student mothers can find them easily and join if support is required. If universities are participating, students will take the support groups seriously and will find it easier to join. This will demonstrate that the university is concerned with the needs and academic well-being of student mothers. Student mothers’ support groups could assist these students to excel academically. Combining parenting and education is difficult, but with the right support, they should be able to balance their various responsibilities. A national education policy should require universities to adopt resources or specifically designed programmes to promote better support for student mothers. For example, universities should provide affordable daycare for student mothers to leave their children while attending classes, reducing the time spent taking and picking up children from a daycare that is far from the university. Furthermore, each department at the university should assign tutors that will be available every day to assist student mothers who missed lectures for a variety of reasons relating to their obligations of childcare.
